# Health Literacy and Task Environment Influence Parents' Burden for Data Entry on Child-Specific Health Information: Randomized Controlled Trial

**DOI:** 10.2196/jmir.1612

**Published:** 2011-01-26

**Authors:** Stephen C Porter, Chao-Yu Guo, Janine Bacic, Eugenia Chan

**Affiliations:** ^4^Division of Developmental MedicineDepartment of MedicineChildren's Hospital BostonBoston, MAUnited States; ^3^Clinical Research ProgramChildren's Hospital BostonBoston, MAUnited States; ^2^Department of PaediatricsUniversity of TorontoToronto, ONCanada; ^1^Division of Paediatric Emergency MedicineThe Hospital for Sick ChildrenToronto, ONCanada

**Keywords:** Medical history-taking, pediatrics, health literacy, computer literacy, task performance and analysis, information dissemination, health records, personal

## Abstract

**Background:**

Health care systems increasingly rely on patients’ data entry efforts to organize and assist in care delivery through health information exchange.

**Objectives:**

We sought to determine (1) the variation in burden imposed on parents by data entry efforts across paper-based and computer-based environments, and (2) the impact, if any, of parents’ health literacy on the task burden.

**Methods:**

We completed a randomized controlled trial of parent-completed data entry tasks. Parents of children with attention deficit hyperactivity disorder (ADHD) were randomized based on the Test of Functional Health Literacy in Adults (TOFHLA) to either a paper-based or computer-based environment for entry of health information on their children. The primary outcome was the National Aeronautics and Space Administration Task Load Index (TLX) total weighted score.

**Results:**

We screened 271 parents: 194 (71.6%) were eligible, and 180 of these (92.8%) constituted the study cohort. We analyzed 90 participants from each arm. Parents who completed information tasks on paper reported a higher task burden than those who worked in the computer environment: mean (SD) TLX scores were 22.8 (20.6) for paper and 16.3 (16.1) for computer. Assignment to the paper environment conferred a significant risk of higher task burden (F_1,178_ = 4.05, *P* = .046). Adequate literacy was associated with lower task burden (decrease in burden score of 1.15 SD, *P* = .003). After adjusting for relevant child and parent factors, parents’ TOFHLA score (beta = -.02, *P* = .02) and task environment (beta = .31, *P* = .03) remained significantly associated with task burden.

**Conclusions:**

A tailored computer-based environment provided an improved task experience for data entry compared to the same tasks completed on paper. Health literacy was inversely related to task burden.

**Trial registration:**

Clinicaltrials.gov NCT00543257; http://www.clinicaltrials.gov/ct2/show/NCT00543257 (Archived by WebCite at http://www.webcitation.org/5vUVH2DYR)

## Introduction

To improve children’s health, effective disease management in attention deficit hyperactivity disorder (ADHD) requires iterative data exchange between pediatric health providers and parents of affected children [1,2]. The classic model of office-based and paper-driven information exchange with the physician as the locus of control often fails to gather data needed for ADHD care [3,4]. Health care systems increasingly rely on technology to organize and deliver care while, at the same time, expecting patients to take on more responsibility for chronic disease management [5,6].

Pediatric providers rely on parents of children with ADHD to report on changes in the child’s health status in order to make treatment decisions [1,2,7]. Parent-provided data on child behaviors and medication use is the first and most elemental information task in a series of data exchanges between a parent and a pediatric health provider that result in health-promoting actions in ADHD. Design of novel, patient-driven systems that support iterative reporting of health information requires better understanding of how parents experience the process of data entry in a single episode [8-10]. To date, no published research has reported on patients’ experience of data entry or identified parent-specific traits or skills that affect task burden related to electronically mediated health communication.

To inform the development of personal health records (PHRs) that invite longitudinal engagement [11,12], and to better understand factors relevant to parents’ successful data entry of information key to ADHD management, we designed a clinical trial to explore the burden experienced by parents during data entry efforts in paper-based and computer-based environments. In addition, we explored health literacy as a parent-specific variable and its impact across task environments [13-15].

The specific aims of this project were to determine (1) the variation in burden imposed on parents by data entry efforts across paper-based and computer-based environments, and (2) the impact, if any, of parents’ health literacy on the task burden experienced across those environments.

## Methods

### Overview

We completed an unblinded, randomized controlled trial of patient-completed data entry tasks using paper-based and computer-based environments to investigate the task burden experienced by parents. Parents of children with ADHD were randomized on the basis of their score on the Test of Functional Health Literacy in Adults (TOFHLA) to provide information on their children’s behaviors, prescribed medications, and potential side effects to medications using either commonly used structured paper forms or a computer-based data entry interface designed to capture the same scope of content. The Committee on Clinical Investigation (Children’s Hospital Boston, Boston, MA, USA) approved the study protocol and the trial was registered. 

### Participants

We recruited English-speaking and Spanish-speaking parents of school-aged children with ADHD. To be eligible for the study, the parent confirmed the following: the child’s age between 5 years and 12 years, that a physician had diagnosed the child with ADHD, that the child resided primarily with the parent, that the parent was the person who managed the child’s health, and that the child was taking or had recently (within the last 4 months) been taking a prescription medication to treat ADHD. Exclusion criteria were the parent’s report of any of the following diagnoses in their child: autism, pervasive developmental disorder, Asperger’s disorder, bipolar disorder, or mental retardation. These criteria were intended to create a study cohort that best resembled a community-based sample of parents caring for a child with ADHD whose disease could be reasonably managed by a primary care provider, and for whom standard forms used in ADHD care for the tracking of symptoms and side effects for medications would be appropriate.

We recruited parents from the greater Boston metropolitan area to participate during a 20-month study period from 2007 to 2009. Outreach efforts for recruitment included newspaper advertisements, letters sent from pediatric practices to inform parents of children receiving care at those practices of our study, emails and listserv postings via parent-support groups specific to ADHD, flyers and brochures displayed and/or handed out at community health centers, adult education centers, child care centers, and other community-based organizations where parents of children with ADHD might visit for services or support. To facilitate recruitment of parents with lower literacy, all materials were developed according to plain-language standards, and personal contact with parents was emphasized among those facilitating our outreach. To facilitate recruitment of Spanish-speaking parents, Spanish-language advertisements were placed in community papers, and Spanish-language materials were used at clinical and community sites where Spanish-speaking parents were known to receive services.

### Consent and Randomization

Parents who indicated interest in the study were screened and completed a stepwise process of consent that included viewing a video explaining the study, discussing the study with research staff, reviewing a one-page plain-language document describing major features of the study and privacy laws, and verbally acknowledging that any questions they had were answered and they wished to proceed with enrollment. 

Prior to randomization, each parent completed the full TOFHLA, a literacy instrument that has been validated in English-speaking and Spanish-speaking populations [16]. The TOFHLA produces a scaled score ranging from 0 to 100 that categorizes functional literacy into three groups: inadequate, marginal, and adequate. Parents were assigned to a “lower literate” group (who scored inadequate/marginal on the TOFHLA) and a “literate” group (who scored adequate on the TOFHLA). Based on this group assignment, each parent was randomized through a mixture of permuted blocks with the goal of equal distribution of literacy levels across the two treatment arms (paper-based tasks vs computer-based tasks). A serially numbered sequence of study IDs with assignments to the two trial arms grouped in randomly permuted blocks of 2, 4, and 6 was generated. This procedure ensured approximate balance between the study arms at any point in time and prevented inadvertent or deliberate bias on the part of those conducting enrollment. 

### Study procedures

Study procedures were completed at a location of the parent’s choosing, with the intention that a majority of parents would prefer to complete tasks in a familiar environment of their own home or a nearby location. In theory, the site where health data tasks related to chronic disease management are completed would mimic where the majority of observations and decisions are made – namely, everyday familiar environments such as the home or nearby community sites.

### Primary study procedures

**Figure 1:**
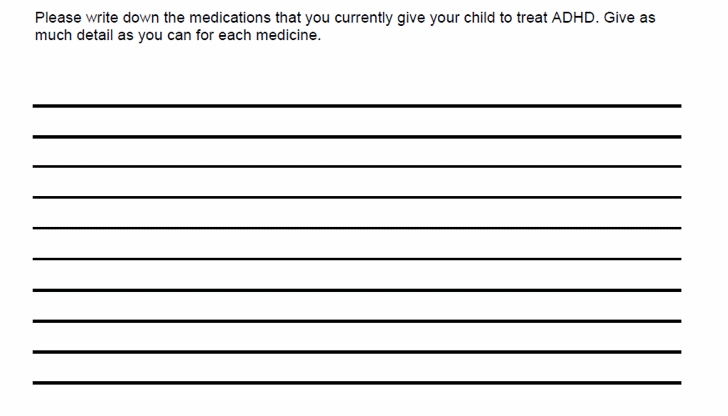
Single-page, paper-based request for information on current medications

Parents randomized to paper first were handed an envelope containing three forms with written instructions for completion. Forms were either in English or Spanish according to the language the parent stated they used in health communication. The parent was told that “these forms are ones similar to what a doctor’s office might send you and ask that you fill out before your next appointment. Please open the envelope and fill out the forms to the best of your ability.” Forms in the envelope were the National Initiative for Children’s Healthcare Quality (NICHQ) Vanderbilt parent assessment form, a medication side-effects inventory, and an open-ended request for information on current medications (Figure 1) [17]. All forms were printed in black and white on 8.5 × 11 inch paper. The research assistant observed parents’ effort with the paper forms and timed the process of data entry but did not provide interpretation of content. 

**Figure 2:**
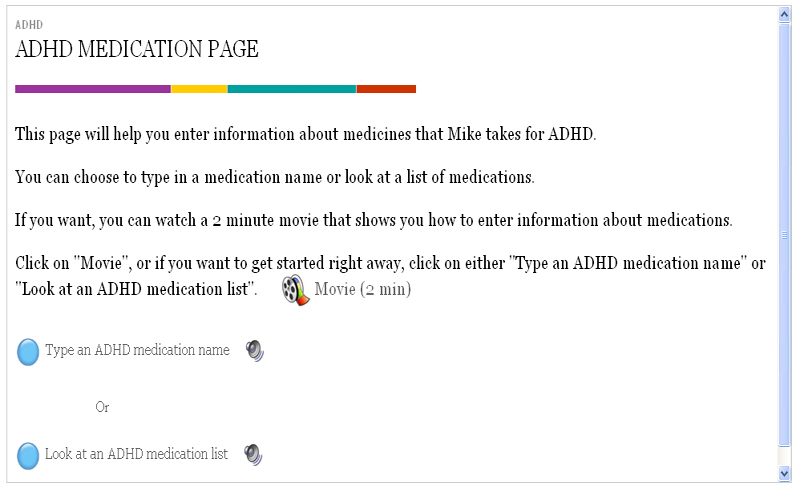
Screen shot of the computer application that guides parents’ entry of medication-specific data

Parents randomized to computer first were introduced to a laptop computer running the ADHD data entry application [18]. The research assistant supervised the parent in completing a log-in procedure that brought up the introductory screen for the ADHD application. At this point, the parent was instructed to follow the directions on-screen and complete the work on their own. The content of computer-based tasks mirrored the content of the paper-based forms, but the structure and workflow on the computer were designed to provide the parent with a guided experience that facilitated comprehension and successful completion of each task (see Figure 2 and Multimedia Appendix 1). The computer application was the end result of a user-centered design process whose goal was the creation of an electronic environment usable by parents with varied technology-specific skills and educational experience [18]. Prerecorded videos were available to help parents who had questions on navigation or content-specific tasks. On-screen navigation required the use of the mouse and familiarity with the scrollbar for vertical movement through displayed content. The research assistant observed the parents’ effort with the computer but did not provide interpretation of content or act as a “help desk” in giving technical assistance. 

After completion of either paper-based tasks or computer-based tasks, each parent was then administered the National Aeronautics and Space Administration Task Load Index (NASA TLX) [19]. The NASA TLX is a multidimensional rating procedure that provides an overall workload score based on a weighted average of ratings on six subscales: Mental Demands, Physical Demands, Temporal Demands, Own Performance, Effort, and Frustration. The degree to which each of the six factors contributes to the workload score is determined by the subject’s responses to pairwise comparisons between the six factors. Magnitude ratings on each subscale are obtained after completion of task performance. Visual analog scales are used to capture subjects’ ratings of task difficulty. The NASA TLX demonstrates content and construct validity and is widely used in human performance studies [20]. A Spanish-language version of the NASA TLX was developed for this study via a process of translation-back translation to ensure that the Spanish text retained the intent of the original English.

After the first data entry task, each parent completed a series of surveys that included questions on how they perceived the task, demographics, technology-specific experience, prior use of health-related forms, and information about their child’s ADHD care. After all surveys were finished, the parent was asked to complete the data entry task using the alternative task environment to which they were not randomized first. 

### Outcomes and Definitions

The primary outcome was the NASA TLX total weighted score.

**Figure 3:**
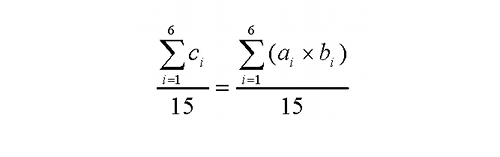
Equation used to calculate the total Task Load Index weighted score

Calculation of the total weighted score for the NASA TLX combines the tally of the number of times a given domain was judged more important to the task experience in pairwise comparisons with the quantification of each domain’s actual burden using a visual analog scale [19]. Each total score is the product of the tally and raw rating (*c_i_* = *a_i_* x *b_i_i* = 1,2,...,6). The weighted rating is the sum of adjusted ratings divided by 15, as the equation in Figure 3 shows.

Secondary outcomes were domain-specific task load, the rank order of task domains, and parental preference for task environment.

### Statistical Methods

The primary unit of analysis was the parental participant. Analyses were completed using an intention-to-treat approach. Sample size for the trial was based on a priori assumptions regarding parents’ accuracy and completeness in report of clinical data, and did not rely on assumptions for NASA TLX scores. The trial met its predetermined sample size of 180 subjects. The primary outcome of the weighted NASA TLX was examined in the normalized format for the primary analysis and the raw score for the secondary analysis.

Since the NASA TLX total weighted score was skewed and the generalized linear model requires the outcome variables to be normally distributed, the weighted score was normalized using the SAS procedure PROC RANK, which computes normal scores, and the resulting weighted scores appeared to be normally distributed with mean equal to 0 and standard deviation equal to 1.

Both crude and adjusted association between the normalized score and task environment were calculated. In the multivariable regression model, covariates included health literacy, years since child’s diagnosis, and the parents’ gender, educational level, race, acculturation, comfort with computers, frequency of computer use, experience in Internet purchasing, experience with paper health forms, and comfort with ADHD terms.

For secondary analysis, nonparametric methods were implemented. The Wilcoxon rank sum test was used to determine whether domain-specific score was associated with task environment, since we only focused on the crude association. The chi-square test was used to determine whether the rank order of task domains or parental preference for task environment was associated with task environment.

All analyses were completed using SAS version 9.1 (SAS Institute Inc, Cary, NC, USA). Tests with the significance level of 5% were considered.

## Results

**Figure 4:**
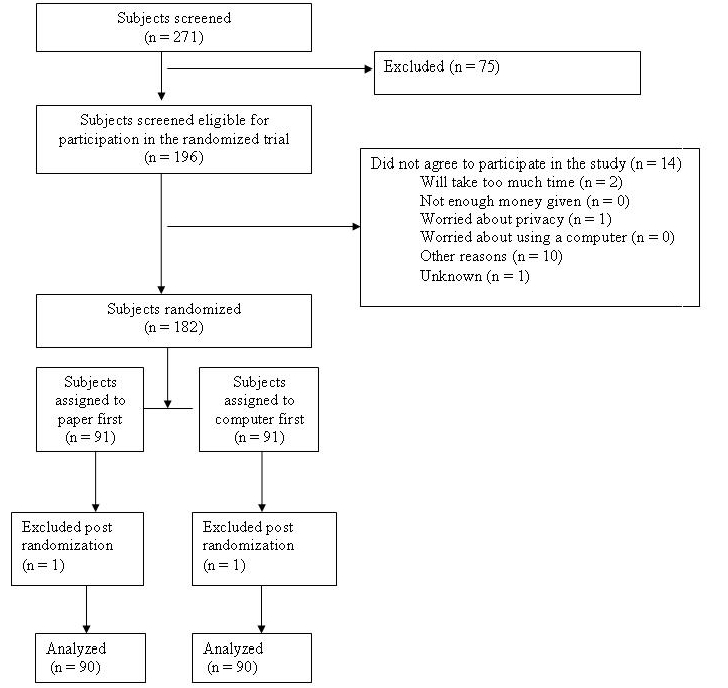
Flowchart of the screening and enrollment process

We recruited and enrolled parents of school-aged children with ADHD in our randomized controlled trial of data entry tasks. A total of 271 parents were screened, 194 of 271 (71.6%) were eligible, and 180 of 194 eligible subjects (93%) constituted the trial cohort for analysis. See Figure 4 for a full account of the screening and enrollment process.

### Description of Parental Cohort

Parents in the enrolled cohort were a diverse group of individuals on the basis of education, race, ethnicity, and experience with the topic of ADHD. Overall, the majority of parents reported exposure to and comfort with the use of computers, including how to navigate the Internet. Table 1 shows the distribution of parents’ characteristics across the randomized groups.

**Table 1:** Baseline characteristics according to randomization

Characteristic		Randomization	
		Paper	Computer
Number of subjects		90	90
TOFHLA^a^ score, mean (SD)		90.90 (9.12)	92.09 (8.92)
**TOFHLA category, n (%)**			
	Inadequate	1 (1)	2 (22)
	Marginal	4 (4)	3 (3)
	Adequate	85 (94)	85 (94)
**Gender, n (%)**			
	Male	4 (4)	7 (8)
	Female	86 (96)	83 (92)
**Ethnicity, n (%)**			
	Hispanic or Latino	10 (11)	10 (11)
	Not Hispanic or Latino	65 (72)	70 (78)
	Other	15 (17)	10 (11)
**Race, n (%)**			
	White	42 (47)	48 (53)
	Black	25 (28)	25 (28)
	More than one race	9 (10)	2 (2)
	Other	14 (16)	15 (17)
**Education level, n (%)**			
	Some grade school/some high school	8 (9)	9 (10)
	Graduated from high school/GED^b^	18 (20)	12 (13)
	Some college or vocational school beyond high school	29 (32)	26 (29)
	Graduated from 2-year or 4-year college	20 (22)	25 (28)
	Post-college graduate courses or degree	15 (17)	18 (20)
**Comfort with ADHD^c^ words, n (%)**			
	Very uncomfortable	8 (9)	12 (13)
	Uncomfortable	6 (7)	8 (9)
	No opinion	6 (7)	10 (11)
	Comfortable /very comfortable	70 (78)	60 (67)
**Experience with paper ADHD form, n (%)**			
	Yes	80 (89)	80 (89)
	No	10 (11)	10 (11)
**Comfort with technology, n (%)**			
	Very uncomfortable	12 (13)	12 (13)
	Uncomfortable	3 (3)	6 (7)
	No opinion	13 (14)	5 (6)
	Comfortable	14 (16)	24 (27)
	Very comfortable	48 (53)	43 (48)

**Years since child’s diagnosis (n, %)**			
	<1	21 (21)	23 (26)
	1-5	54 (60)	48 (53)
	>5	15 (17)	19 (21)

^a^ TOFHLA: Test of Functional Health Literacy in Adults.

^b^ GED: general educational development.

^c^ ADHD: attention deficit hyperactivity disorder.

### Impact of Task Environment on Task Experience

**Figure 5:**
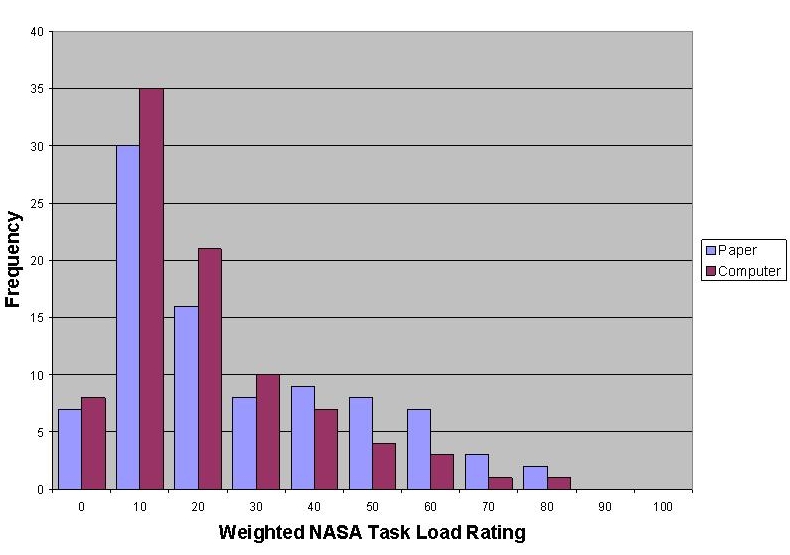
Distribution of National Aeronautics and Space Administration Task Load Index (NASA TLX) score by task environment

Parents who completed the information tasks in the paper environment reported higher task burden than those who worked in the computer environment: mean (SD) of TLX score for paper was 22.8 (20.6) and for computer was 16.3 (16.1). In a generalized linear model with TLX score as the dependent variable, assignment to the paper environment conferred a significant risk of higher task burden (F_1,178_ = 4.05, *P* = .046). Figure 5 graphically displays the distribution of TLX scores across the two task environments.

### Impact of Health Literacy

Health literacy as measured by the TOFHLA showed an inverse relationship to task experience. Across all subjects, adequate literacy was associated with lower task burden (decrease in burden score of 1.15 SD, *P* = .003). Subjects who scored adequate on the TOFHLA were significantly more likely to experience lower task burden in the paper environment (decrease in score of 1.63 SD, *P* < .001). This differential effect for paper-based tasks was not as prominent for parents using the computer. In the computer environment, subjects who scored adequate had a lower but nonsignificant difference in burden score than those who scored marginal/inadequate for literacy (decrease in score of 0.68 SD, *P* = .11). 

In a generalized linear model that controlled for task environment, subjects who scored adequate for literacy were significantly more likely to experience lower task burden (decrease in score of 1.15 SD, *P* < .001).

### Analysis of Health Literacy’s Adjusted Association With Task Burden

A multivariable model was constructed to further explore the strength of health literacy’s association with task burden as the dependent variable, and health literacy modeled as a continuous covariate (see Table 2). After adjusting for years since their child’s diagnosis and the parents’ gender, educational level, race, ethnicity, acculturation, comfort with computers, frequency of computer use, use of the Internet to make purchases, experience with paper health forms, and comfort with ADHD terms, parents’ TOFHLA score remained significantly associated with task burden (F_1,178_ = 5.4, *P* = .02). Of note, task assignment also remained significant in this model (F_1,178_ = 4.6, *P* = .03). Data entry in the computer environment and a higher TOFHLA score both favored an improved task experience by parents. 

**Table 2:** Crude and adjusted linear regression models with task burden as outcome

Variable name		Beta	Standard error	*P*-value
**Crude model**				
	Task environment (paper vs computer)	.294	0.146	.04
	Health literacy score	-.0293	0.008	.0003
**Multivariable model**					
	Task environment (paper vs computer)	.310	0.145	.03
	Health literacy score	-.024	0.010	.02
	Internet purchase experience (yes vs no)	.354	0.188	.06
	Generic computer experience (yes vs no)	-.424	0.229	.06
	Comfort with ADHD^a^ words (yes vs no)	-.202	0.176	.25
	Time since diagnosis	-.072	0.069	.30
	Education (high school yes vs no)	.188	0.197	.34
	ADHD paper form experience (yes vs no)	-.213	0.239	.37
	Race (white vs others)	-.081	0.165	.62
	Born in the United States (yes vs no)	-.108	0.239	.65
	Parent gender (male vs female)	.086	0.304	.78
	Comfort with computer use (yes vs no)	-.039	0.175	.82

^a^ ADHD: attention deficit hyperactivity disorder.

### Secondary Analysis of Individual TLX Domains

The six domains that constitute the NASA TLX (mental demand, physical demand, temporal demand, effort, frustration, performance) were explored for their individual relationships to task environment and to the health literacy of subjects. In all domains except physical demand, the paper environment was associated with higher task burden, although no significant differences between medians were found. Table 3 highlights the details of this comparison.

**Table 3:** Comparison of task burden for individual domains in paper versus computer environments

Domain	Paper		Computer		*P*-value
	TLX^a^ score	IQR^b^	TLX score	IQR	
Mental demand	67.5	(20-195)	50	(15-120)	.11
Physical demand	0	(0-0)	0	(0-10)	.12
Temporal demand	2.5	(0-40)	0	(0-25)	.37
Effort	50	(5-180)	40	(0-120)	.19
Frustration	0	(0-20)	0	(0-10)	.45
Performance	2.5	(0-75)	0	(0-30)	.06

^a^ TLX: National Aeronautics and Space Administration Task Load Index.

^b^ IQR: interquartile range.

We also explored which domains were ranked by subjects as the most and least important contributors to burden across the two environments. Subjects ranked mental demand as the most influential contributor to burden across both task environments, with 47 subjects in the paper environment and 43 subjects in the computer environment ranking it first. Subjects also reported that both task environments required significant effort, with 35 subjects in the paper environment and 37 subjects in the computer environment ranking it first. Least influential domains across both environments were physical demand (9 paper, 12 computer) and frustration (10 paper, 14 computer). We found no statistically significant differences when comparing the summative rank for each domain between those assigned to paper and those assigned to computer (data not shown).

We further investigated the relationship between health literacy and the rank order of domains judged to be most and least important to subjects when data from both task environments was combined. For mental demand and effort, subjects who scored adequate on the TOFHLA were no more likely than those who scored marginal/inadequate to rank either domain as most important (mental demand, 88/170 vs 2/10, *P* = .10; effort 68/170 vs 4/10, *P* = 1.0). Notably, subjects who scored adequate on the TOFHLA were more likely than those who scored inadequate/ marginal to rank the domain of frustration as least important (115/170 vs 3/10, *P* = .03). There was no significant difference in ranks for the domain of physical demand by TOFHLA category (data not shown). 

### Parents’ Preferences Regarding Task Environments

After completion of data entry tasks within both paper-based and computer-based environments, parents were surveyed with regard to their preference for which environment they would use if they had to repeat the task in the future. Most (141/180, 78.3%) stated their preference for the computer-based task environment. Table 4 summarizes the reasons given by parents for their preference of task environment for data entry (note that parents were able to choose multiple reasons that explained their preference).

Parents’ preference did not vary based on which task environment they were assigned to first (c^2^_1_ = 180) = 0.10, *P* = .74). Literacy scores varied between parents who preferred the computer environment to paper, but this variation was not statistically significant (mean TOFHLA score for computer (paper) was 94 (77), c^2^_1_ = 180) = 3.5, *P* = .06).

**Table 4:** Number of participants in each group selecting reasons for preference on task environment

Reason	Preference	
	Computer	Paper
It is easier to complete this version	89	10
It is quicker to complete this version	61	10
I feel more comfortable completing this version	29	19
It is easier to read the instructions and questions in this version	25	2
I like writing more than typing, or vice versa	16	3
Other	27^a^	9
Total	141	39

^a^ 4 responses indicate benefit for storage and organization; 4 responses indicate convenience as a benefit; 3 responses indicate benefit for improved quality of record; 3 responses indicate benefit for capacity to edit.

## Discussion

### Principal Results

In this randomized trial, parents completing data entry tasks specific to their child’s ADHD reported superior task experience using a tailored computerized environment compared to using standard paper forms for tracking symptoms, medications, and side effects. The majority of parents preferred the computer environment for the task of entering health information. Notably, working in the computer environment attenuated a disparity with regard to parents’ literacy level and its association with reported task experience. Our results support the contention that a tailored, patient-centered electronic interface provides benefits that lead to patients’ being more willing to re-engage in a subsequent information-giving task – a key construct in optimizing disease control. 

Our study was notable for its examination of health literacy as a predictor and the identification of the independent effect of literacy as a predictor for parents’ report of task experience. Lower health literacy was associated with higher task burden independent of the task environment. Although assignment to the computer environment attenuated this disparity, it was not eliminated completely. Specific attributes of the tailored electronic interface may explain the improvement in disparity compared to paper. These include the interface’s multimedia format with colors and pictures that reinforce the parent-child relationship, the navigational path of a home page with 3 defined steps, and feedback about progress through the application. [14,15,21].

Health literacy remained a significant factor in explaining variation in task experience even after adjustment for variables that account for parents’ technology experience, experience with paper forms, sociodemographic descriptors, and years since child’s diagnosis. This finding reinforces the importance of considering literacy during the design of patient-centered information solutions that include data entry tasks [14]. The work of documenting information is not just an expressive act of communication. It also demands skills in reading, problem solving, and organization as an individual attempts to understand and implement the instructions [22].

### Comparison to Prior Work

Models of technology acceptance have identified important constructs that underlie individuals’ willingness to engage with an electronic interface: these include self-efficacy with regard to technology, as well as perceived ease of use and usefulness [23,24]. The primary outcome of our study, the NASA TLX, summarizes and quantifies each subject’s perceptions of what it took to complete the task and the amount of burden imposed. Importantly, the domains of the TLX include attributes that address both self-efficacy (performance) and ease of use (demand on body, mind, and time). Parents’ endorsement of the computer as the preferred environment if the task is repeated in the future can be viewed as a summative, parent-level view of which environment best fits the repeated work of communicating health data specific to ADHD. 

Our work addresses a debate in the literature as to whether the digital divide negatively affects traditionally underserved patients on the basis of education, income, or literacy. Our findings highlight a benefit of the computer-based environment for lower-literate parents that are in concert with two prior studies; namely, that a tailored computer-based user interface can meet the needs of patients presumed to be challenged by a computer environment due to lack of knowledge, skills, or self-efficacy. Chen and Zhang in their work comparing graphic and text-based user interfaces noted that “novice users” obtained benefit from a more graphic-based approach compared to expert users, who performed equally well in both environments [25]. In addition, research in cancer communication noted that lower-income patients demonstrated higher access rates than more educated subjects to a comprehensive computer-based support system designed for ease of use [26].

Our results reinforce findings from an earlier study that reported how data entry tasks on health topics impose some effort and mental demand on the reporter [27]. We did not find any differences across the task environments with regard to how individual domains contributed to the overall task experience. The parents with adequate literacy were not different from parents scoring marginal/inadequate with regard to how they ranked the domain with the greatest impact on overall burden. 

This investigation of task experience purposefully recruited a community-based sample of parents and studied their data entry efforts in the context of their usual daily environment [28]. Efforts to understand and optimize patient-centered information management strategies require a diverse group of subjects who are completing health tasks at home or a location they frequent in their community – that is, in the physical environment where the task would actually be completed. Although this type of field research introduces variability, as different homes and community locations may introduce different distractions from noise and interruptions, the randomized nature of the trial provides some protection for unmeasured confounders. 

Our results inform development of pediatric-specific electronic solutions that call for parents’ report of data on behalf of their children. Health literacy, independent of other factors, affects the user experience specific to data entry. Structured electronic interfaces that attend to plain-language goals, provide sufficient “help” functionality, and include multimedia strategies for communication of health data have the potential to mitigate disparity on the basis of health literacy.

### Limitations

Several important factors limit our results. We cannot directly address the task burden of longitudinal engagement with a PHR, as we studied only one episode of data entry. Further, the data entry step is only the first of a series of likely interactions that a given patient would have with a PHR, and our findings with regard to task burden for patient-PHR interactions must be viewed conservatively. Our investigation of literacy is limited by the small number of individuals with lower literacy in our recruited cohort. The possibility of a selection bias in our parent sample may persist despite efforts during recruitment to reach eligible parents through social-support networks and community-based service agencies that are not traditional sites of health care delivery. Despite these issues, the results provide a novel perspective on parents’ perception of task burden across two common channels used for health communication: paper and computer. Furthermore, the community-based nature of our study of data entry tasks provides important generalizability. 

### Conclusions

This study suggests that a tailored computer-based environment provided an improved task experience for data entry compared to the same tasks completed on paper. Health literacy was inversely related to task burden. Disparities in burden experienced by parents with lower literacy in the paper environment were attenuated by assignment to completion of tasks using the computer. Health literacy is the most significant predictor of task burden across measured parental characteristics. These findings are relevant to the design and implementation of PHR solutions for pediatric chronic disease where parents’ data entry is a key step in information exchange about their child’s health. 

## Multimedia Appendix 1

Slide 1 displays the one-page unstructured paper form used by parents to document medications. Slides 2-8 display screen shots from the attention deficit hyperactivity disorder (ADHD) application specific to reporting on medications.

## Abbreviations

ADHD: attention deficit hyperactivity disorder
NASA: National Aeronautics and Space Administration
NICHQ: National Initiative for Children’s Healthcare Quality
PHR: personal health record
TLX: Task Load Index
TOFHLA: Test of Functional Health Literacy in Adults
