# Supplementary figures and images for: Health Literacy and Task Environment Influence Parents' Burden for Data Entry on Child-Specific Health Information: Randomized Controlled Trial
Source: J Med Internet Res. 2011 Jan 26;13(1):e13. doi: 10.2196/jmir.1612 (PMC3221334; doi:10.2196/jmir.1612)

## Slide 1
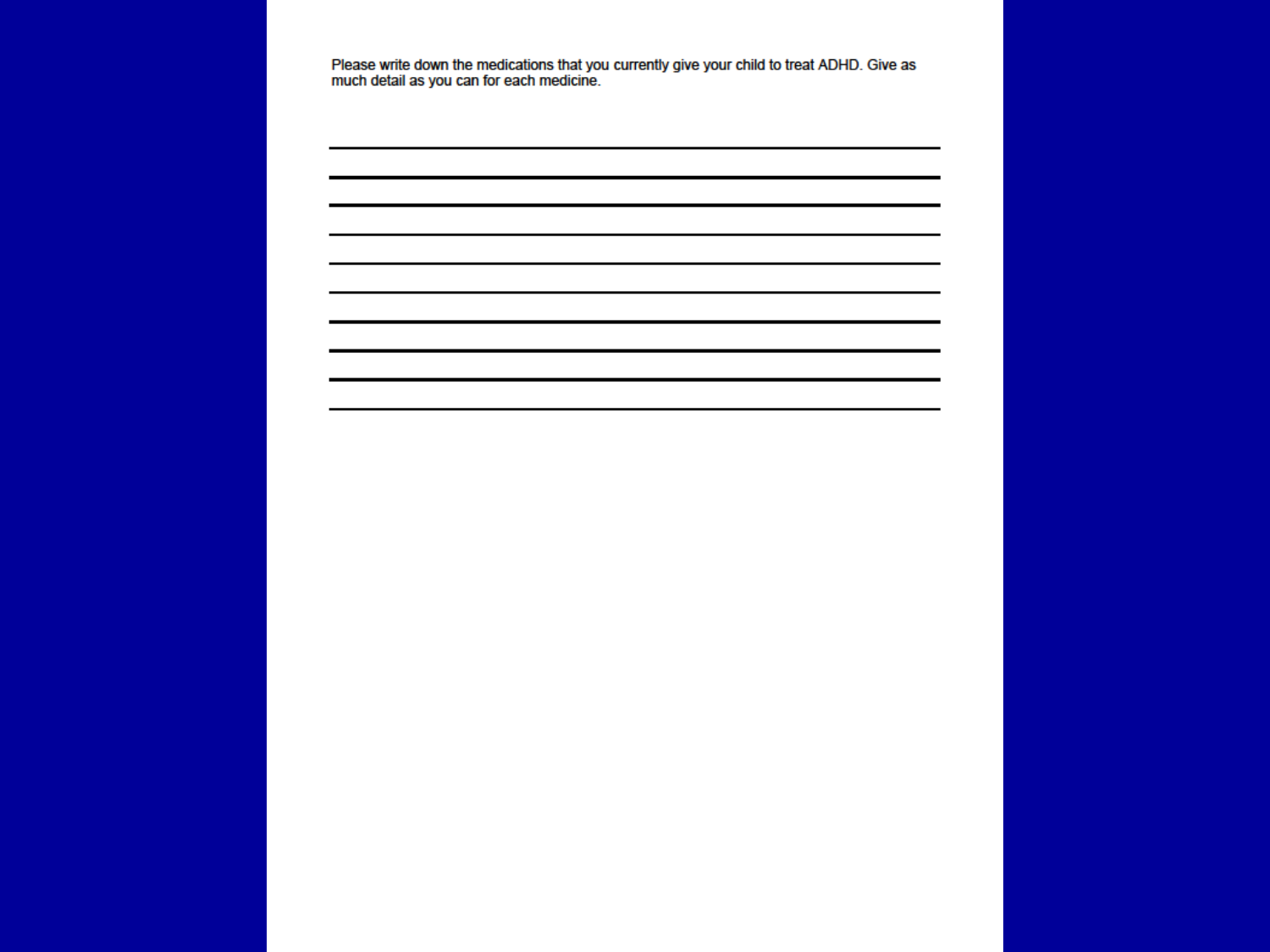

## Slide 2
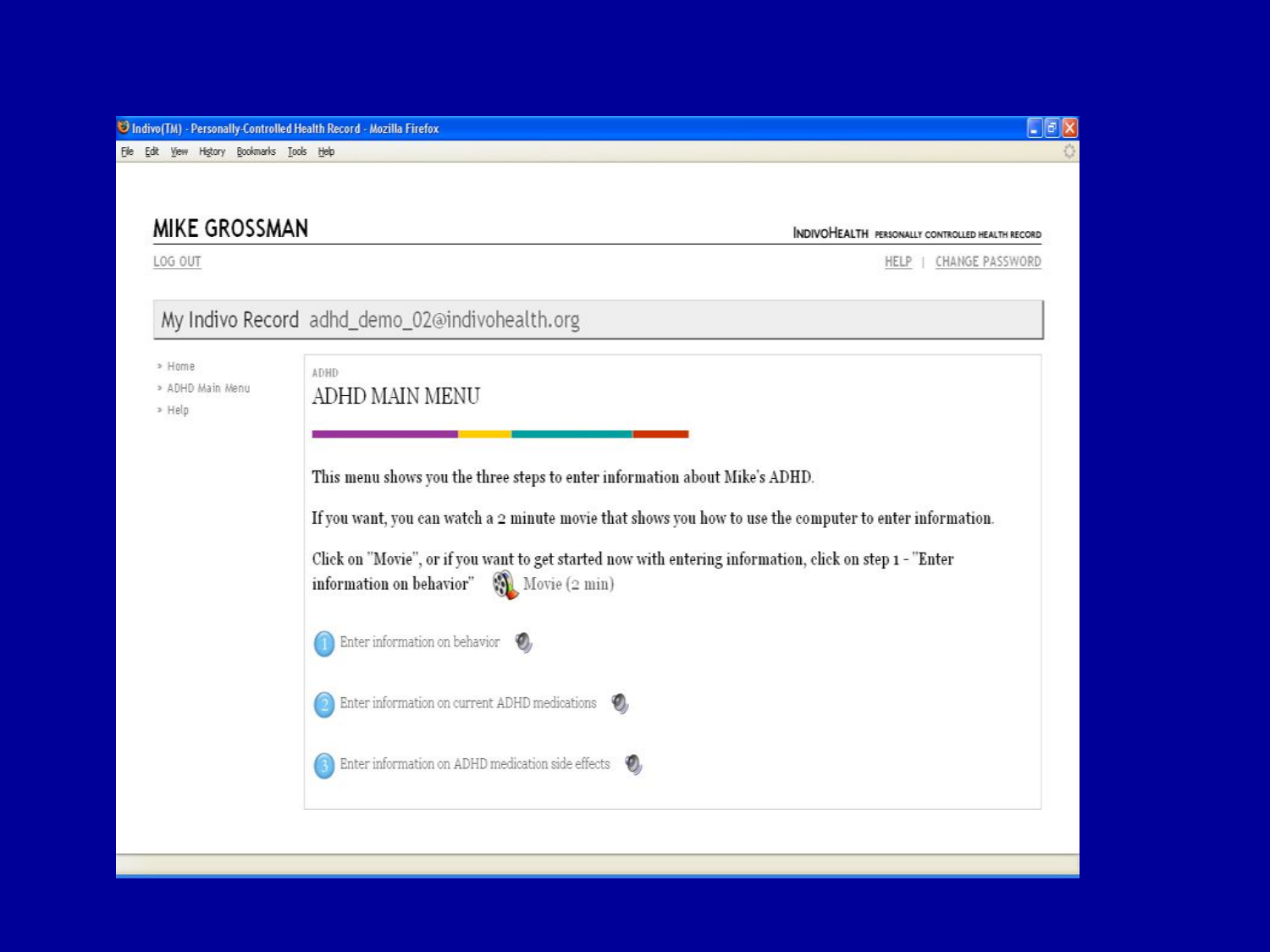

## Slide 3
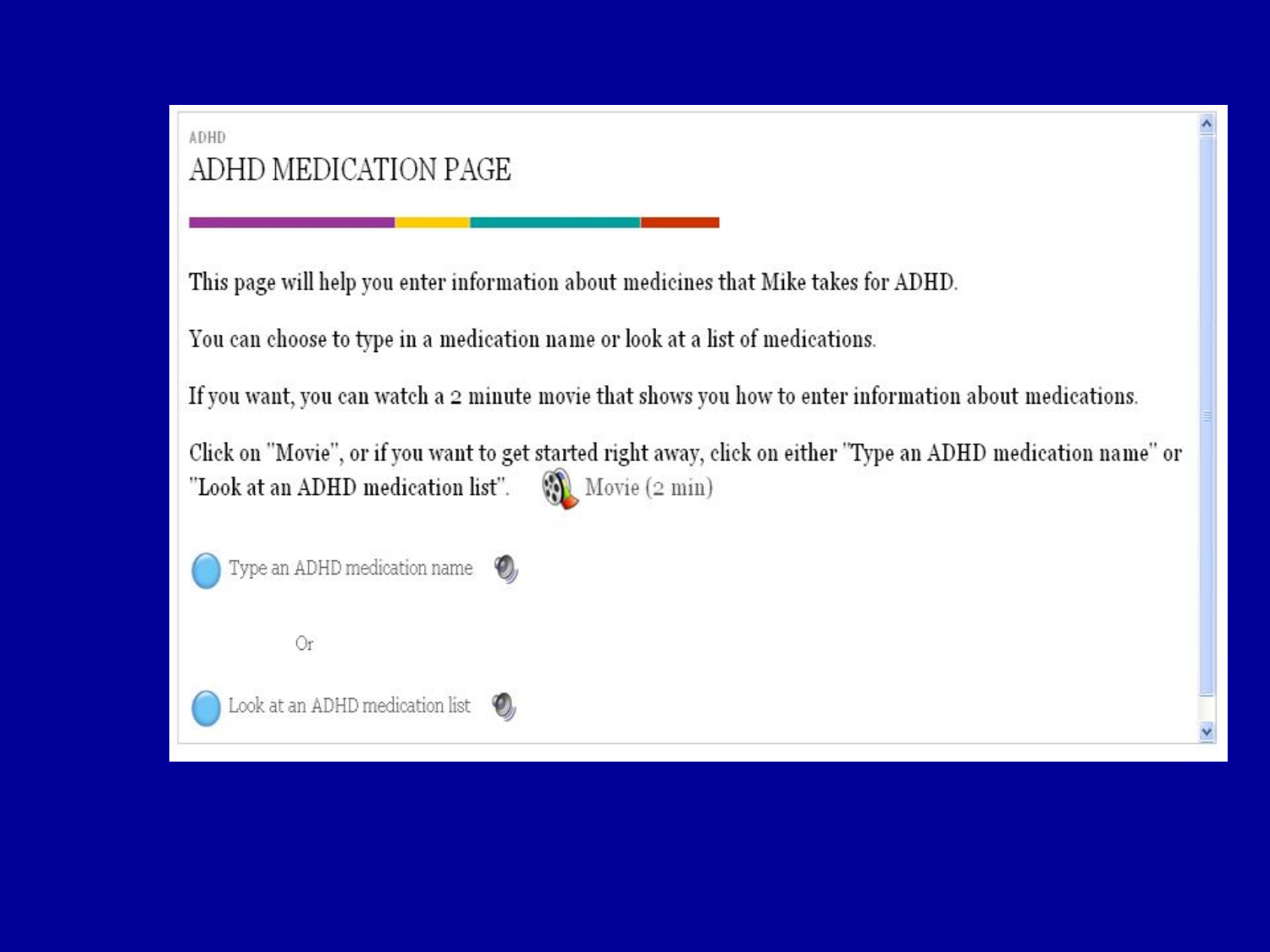

## Slide 4
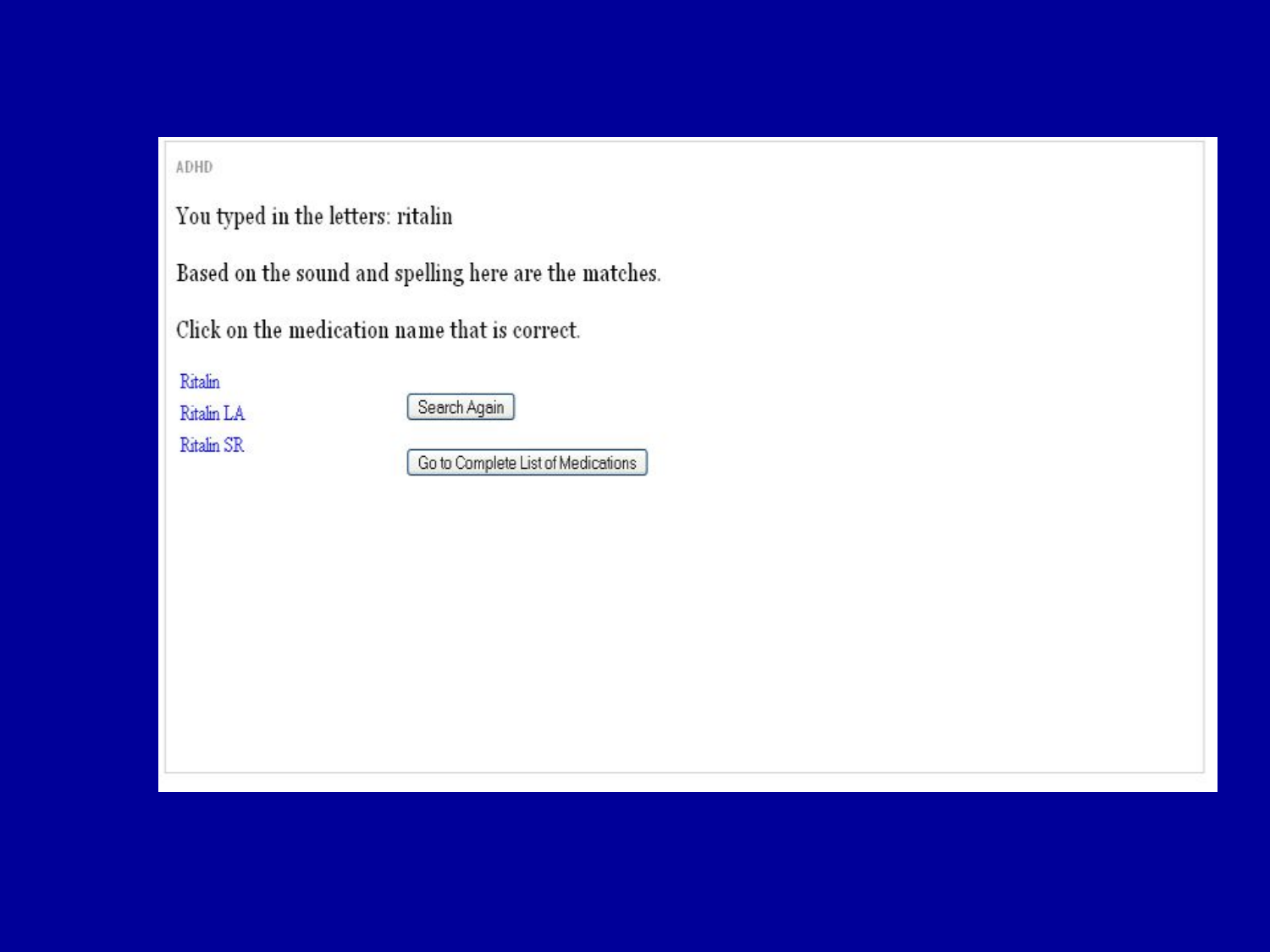

## Slide 5
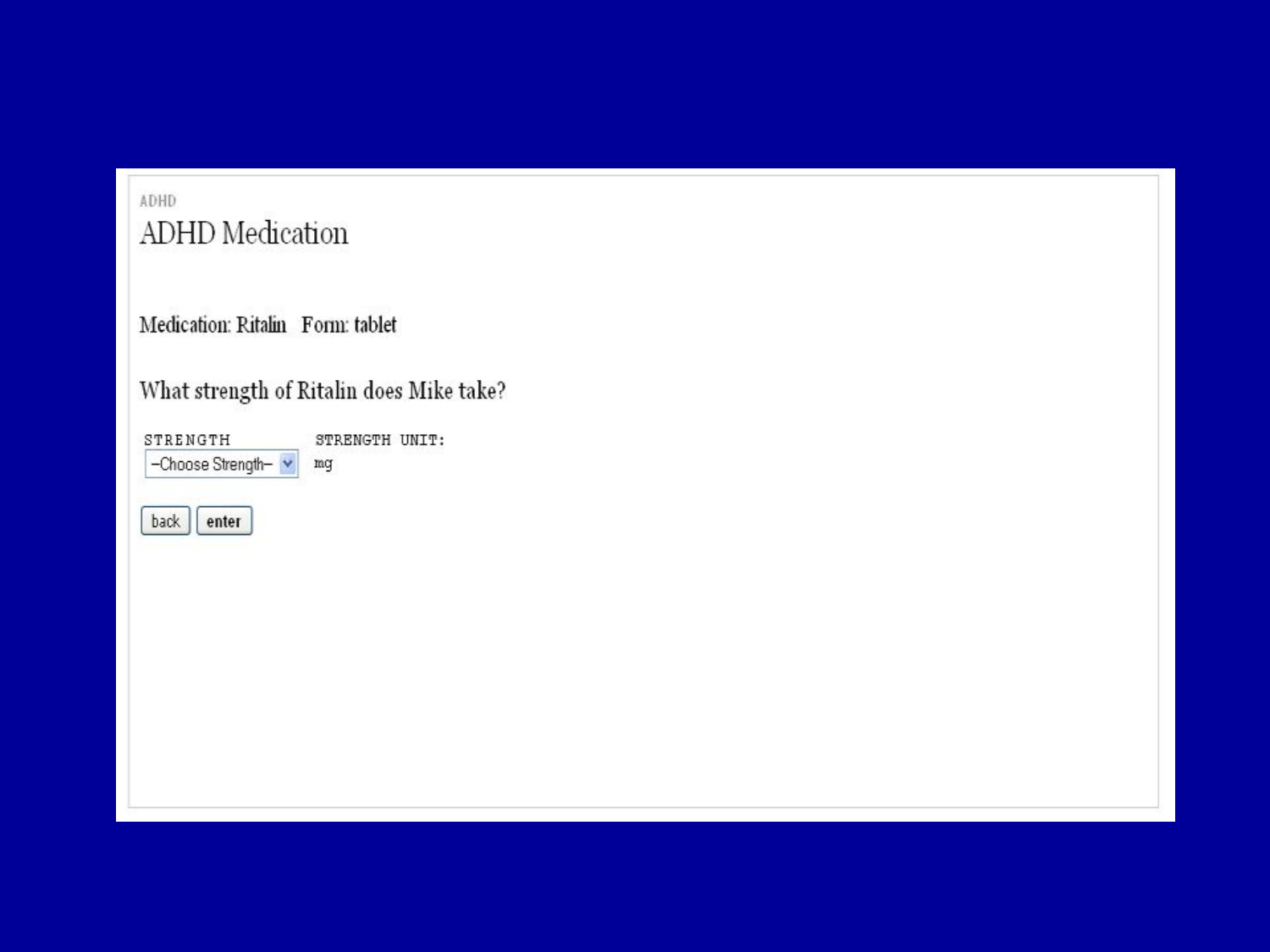

## Slide 6
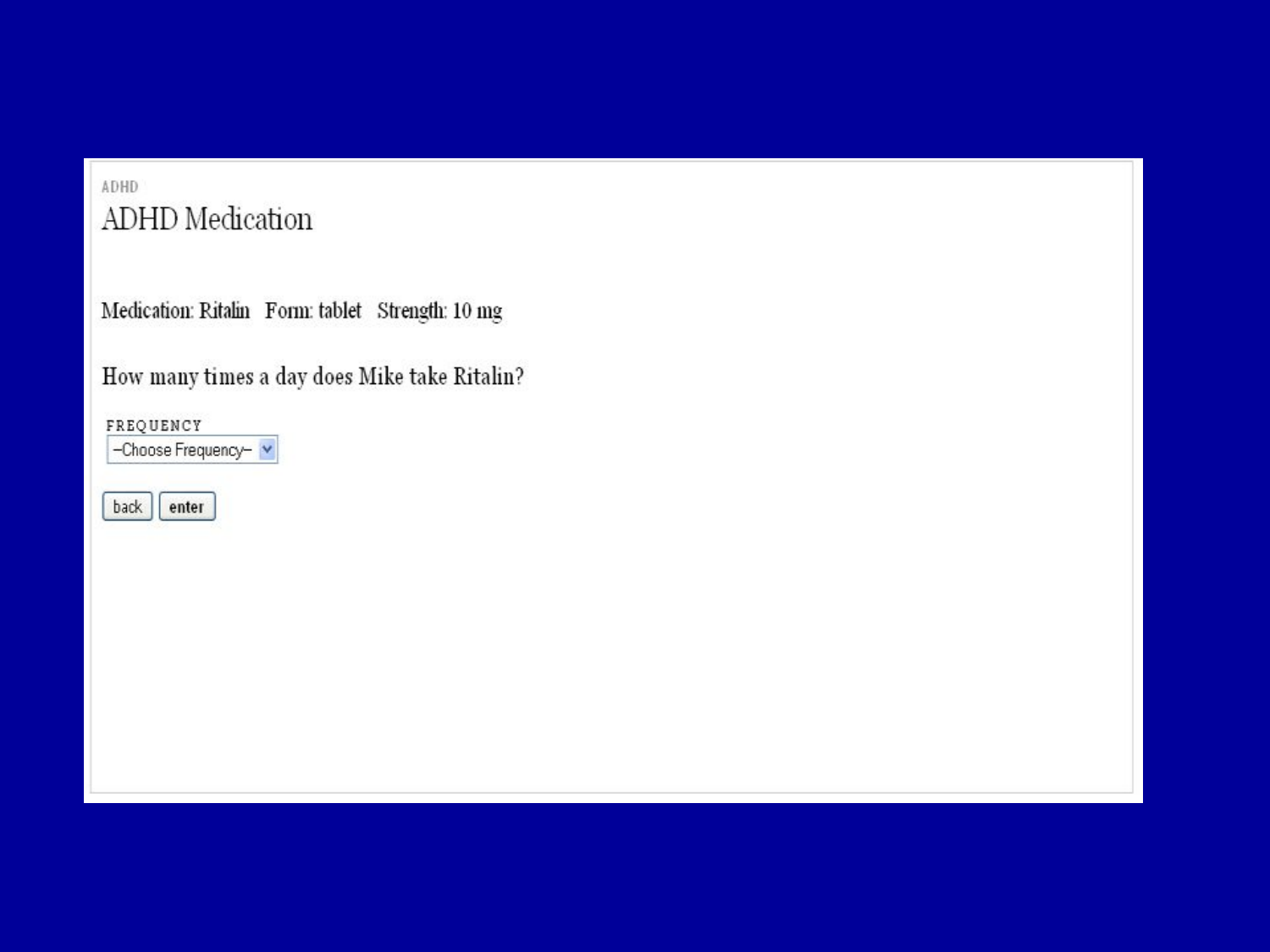

## Slide 7
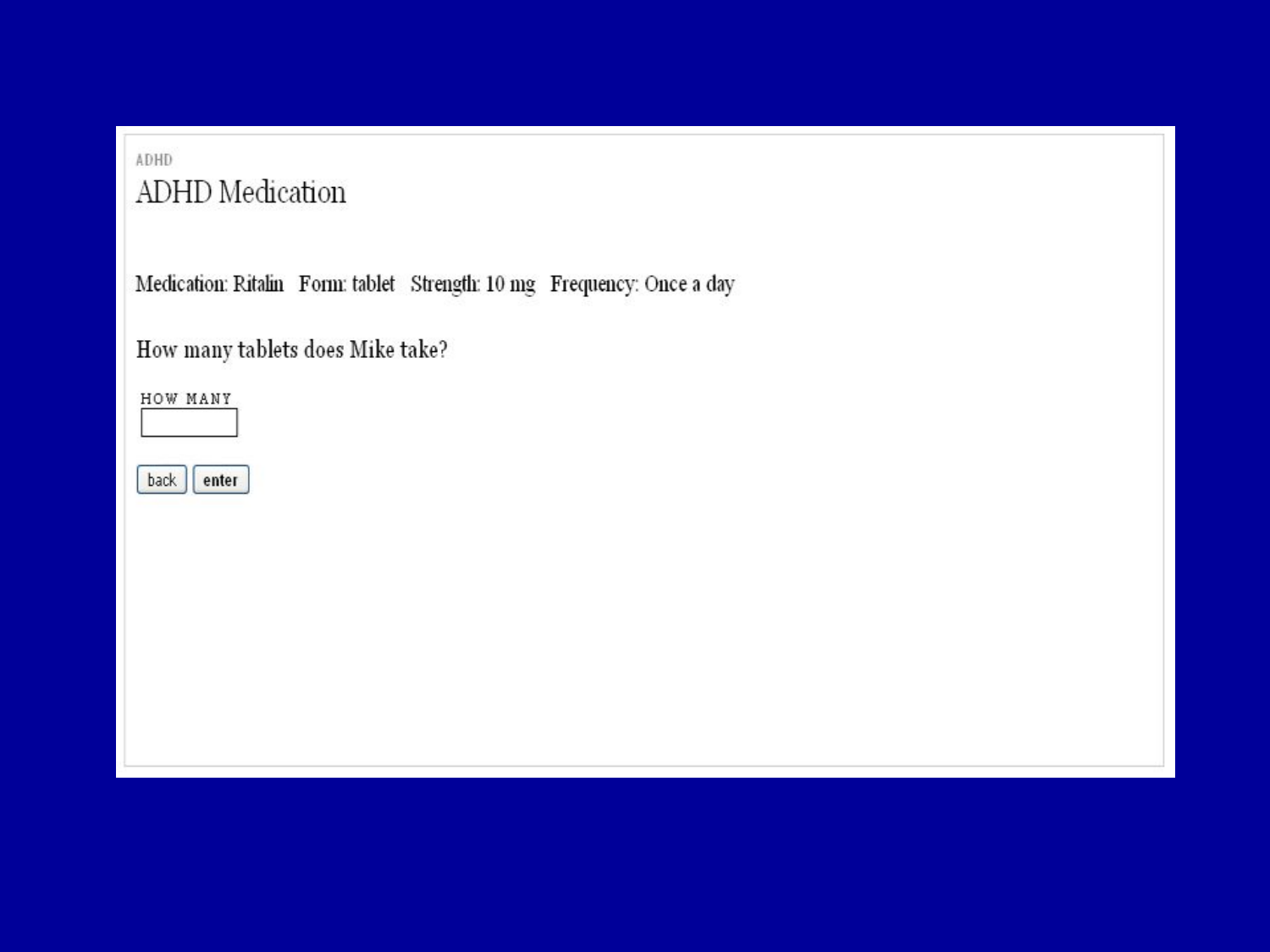

## Slide 8
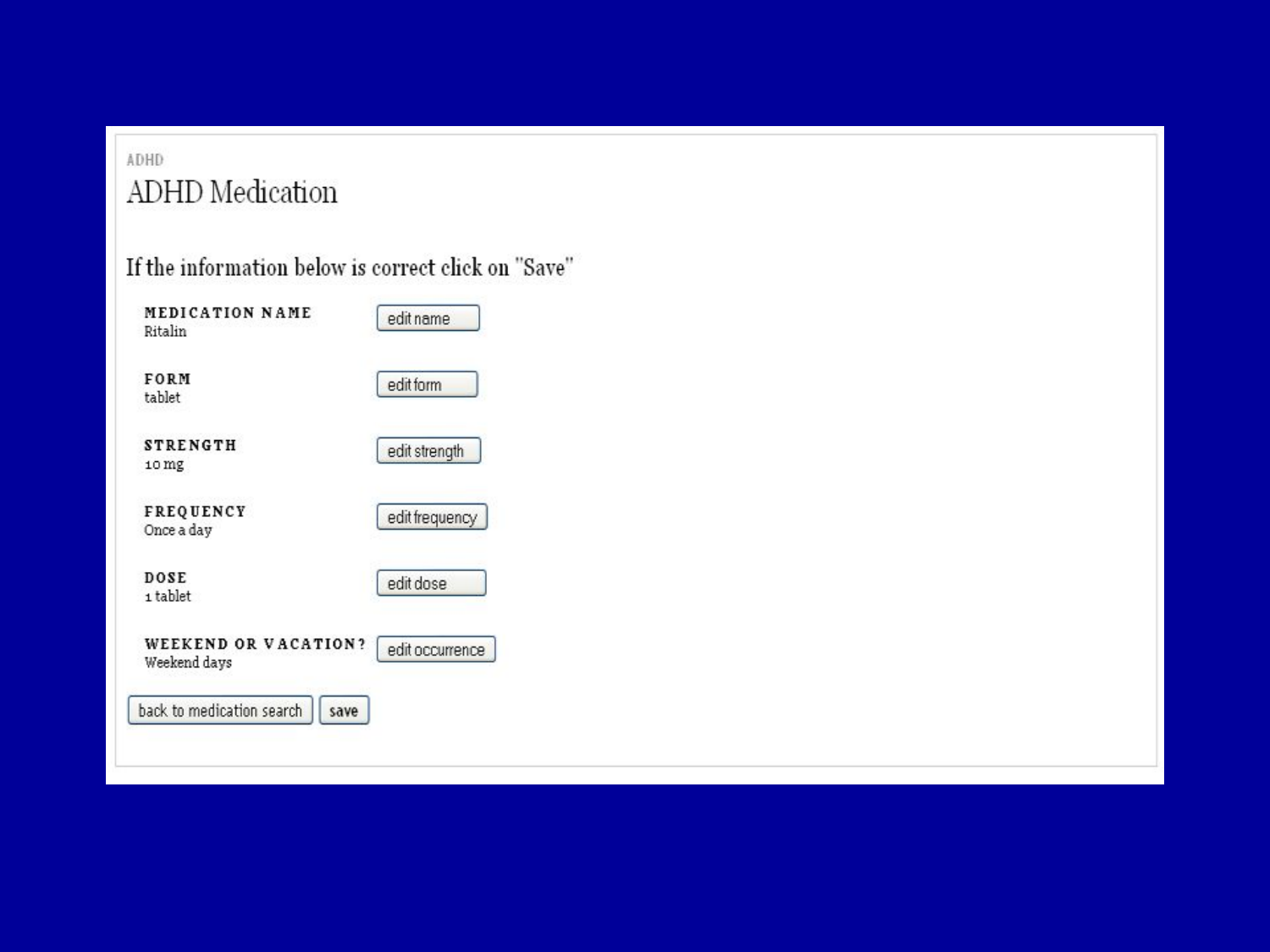

Supplement: Supplementary file 1 [file jmir_v13i1e13_app1.ppt]
